# Pulse pressure is associated with Alzheimer's disease pathology and cognitive decline

**DOI:** 10.1002/alz70857_104595

**Published:** 2025-12-25

**Authors:** Joon Hyung Jung, Nayeong Kong, Seunghoon Lee

**Affiliations:** ^1^ Chungbuk National University Hospital, Cheongju, Korea, Republic of (South); ^2^ Department of Psychiatry, Keimyung University Hospital, Daegu, Korea, Republic of (South); ^3^ Myongji hospital, Hanyang University College of Medicine, Goyang, Korea, Republic of (South)

## Abstract

**Background:**

Aging is accompanied by vascular changes, notably arterial stiffness, which is reflected by pulse pressure (PP). Elevated PP has been linked to cardiovascular and cerebrovascular diseases, as well as cognitive decline and dementia. However, its specific role in Alzheimer's disease (AD) development and progression remains underexplored. This study investigates the association between baseline PP and key AD biomarkers—amyloid‐beta (Aβ) and tau deposition—and cognitive decline.

**Method:**

This study utilized data from 1,690 cognitively unimpaired older adults participating in the Anti‐Amyloid Treatment in Asymptomatic Alzheimer's (A4) and Longitudinal Evaluation of Amyloid Risk and Neurodegeneration (LEARN) studies. Biomarkers were assessed using positron emission tomography (PET) imaging for Aβ and tau (*n* = 411) deposition. Cognitive function was evaluated using the Preclinical Alzheimer Cognitive Composite (PACC). Cross‐sectional analyses were conducted to examine associations, with linear regression adjusted for age, sex, education, and APOE4 status.

**Result:**

We revealed that elevated PP was significantly associated with increased global and inferior temporal tau deposition (standardized β = 0.11, *p* =  0.020; standardized β = 0.12, *p* =  0.012). Moreover, elevated PP was also associated with higher Aβ deposition (standardized β = 0.06, *p* =  0.011) and with lower PACC (standardized β = ‐0.05, *p* =  0.034). APOE4 status moderated the relationship between PP and tau deposition; in APOE4 carriers, higher PP was strongly associated with tau accumulation, while no significant relationship was observed in non‐carriers.

**Conclusion:**

These findings underscore the importance of arterial stiffness as a contributor to AD pathology and lower cognition, particularly in individuals with genetic susceptibility. By revealing how PP interacts with APOE4 to influence tau deposition, this study emphasizes the need for targeted interventions addressing vascular health to mitigate AD progression. Future research should further explore mechanisms linking vascular health and AD biomarkers to refine preventative strategies.

